# Consistent Quadratic Phase Formation in 3D Fast Spin Echo Using Frequency‐Modulated RF Pulses

**DOI:** 10.1002/mrm.70368

**Published:** 2026-04-02

**Authors:** Naoharu Kobayashi, Michael Garwood

**Affiliations:** ^1^ Center for Magnetic Resonance Research, Department of Radiology University of Minnesota Minneapolis Minnesota USA

**Keywords:** fast spin echo, frequency‐modulated RF pulse, inhomogeneous field MRI, quadratic phase

## Abstract

**Purpose:**

Frequency‐modulated (FM) RF pulses achieve broadband excitation with low RF peak power, which is required in MRI with inhomogeneous magnetic fields. However, the quadratic phase generated with FM pulses makes it difficult to use them in fast spin echo (FSE), because even and odd refocused echoes have different spatial phase profiles. In this article, we formulate the condition that can generate a consistent quadratic phase in the two echo components in 3D FSE using FM pulses.

**Methods:**

The consistent quadratic phase condition in 3D FM‐FSE was formulated with the Cayley‐Klein parameters. The B_1_^+^‐dependent phase in FM excitation was compensated by adjusting the initial phase of FM pulses. The consistent quadratic phase in 3D FM‐FSE and the impacts from the B_1_^+^‐dependent phase were simulated with extended phase graph (EPG). The EPG simulation was validated by comparing with experimental results at 3 T. Finally, in vivo brain imaging was conducted with *T*_1_‐ and *T*_2_‐weighted contrasts.

**Results:**

In EPG simulation, a consistent quadratic phase was generated in all echoes in 3D FM‐FSE under the formulated condition. The EPG simulation matched well with experimental results. The B_1_^+^‐dependent phase adjustment on FM pulses improved the magnitude profile of the refocused echoes. Despite the presence of large field inhomogeneity, 3D FM‐FSE achieved in vivo brain imaging with good *T*_1_‐ and *T*_2_‐contrasts.

**Conclusion:**

Achieving consistent quadratic phase in all refocused echoes in 3D FM‐FSE makes it possible to use FM pulses in the non‐adiabatic regime. 3D FM‐FSE is a promising pulse sequence for MRI in inhomogeneous magnetic fields.

## 1 Introduction

Frequency‐modulated (FM) RF pulses produce spin excitation by frequency sweeping across the range of resonance frequencies of interest sequentially in time. Accordingly, by using low peak RF power and long pulse duration, it is possible to excite broadband frequencies with FM pulses. The broadband coverage with FM pulses was previously used in various pulse sequences such as fast spin echo (FSE) [1, 2], missing‐pulse steady‐state free precession [3, 4], and a spatiotemporal encoding method [5], to achieve broadband excitation/refocusing in MRI with inhomogeneous magnetic fields. However, the quadratic phase profile introduced by the FM pulses cannot be refocused using linear gradient magnetic fields applied with the gradient coil sets in MRI systems. Therefore, the quadratic phase is usually removed under specific conditions of FM pulses such as an adiabatic double spin‐echo sequence using a pair of identical refocusing FM pulses [6] and adiabatic spin‐echo sequences using matched excitation and refocusing FM pulses [7, 8, 9].

While such techniques remove the quadratic phase associated with FM pulses, they cannot be applied in the context of FSE with multiple refocused echoes. Accordingly, the previously introduced FSE method using FM pulses creates two types of echoes in the FSE echo train: “FID” and “spectral” echoes [1]. These two echoes show up alternately in the FSE echo train (e.g., odd echoes are “spectral” echoes with the quadratic phase and even echoes are “FID” echoes without the quadratic phase). Thus, it typically uses one of the two echoes [10] or processes the two echoes separately [1]. Additionally, the refocusing FM pulses in the FSE sequence need to be adiabatic (i.e., 180° pulses) to avoid the mixture of these two echo components with inconsistent phase profiles.

In this article, we first formulate the condition where a consistent quadratic phase is generated in even and odd refocused echoes in 3D FSE. Under this condition, the even and odd echoes can be combined without removing the quadratic phase in 3D image reconstruction, because consistent quadratic phase is produced in all refocused echoes. Hence, the refocusing FM pulses in the proposed FM‐FSE sequence are not necessarily adiabatic. The formulated consistent quadratic phase condition was first tested in extended phase graph (EPG) simulation and then experimentally validated on a clinical 3 T MRI scanner. In this study, because the clinical MRI system has a highly homogeneous static magnetic field, we introduced a linear z gradient field as an inhomogeneous magnetic field during the entire FM‐FSE scan. With the developed FM‐FSE sequence, in vivo human brain scans were conducted with *T*_1_‐ and *T*_2_‐weighted contrasts. Finally, performance of 3D FM‐FSE in a nonlinear inhomogeneous field was also tested by setting the Z^2^ shim value of the B_0_‐shim coil set to its maximum in the clinical 3 T MRI system.

## 2 Theory

### 2.1 Quadratic Phase Generated by FM RF Pulses

**FIGURE 1:**
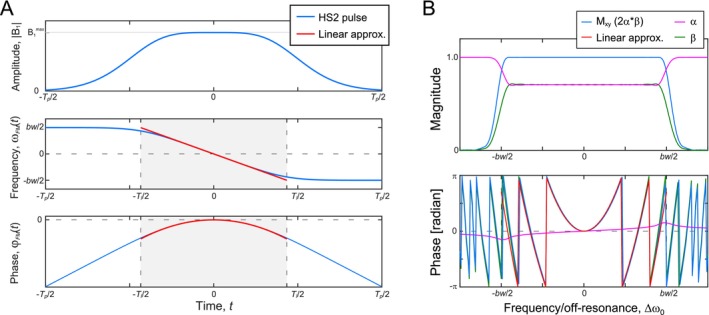
(A) Linear sweep approximation of a flattened hyperbolic secant (HS2) pulse used in this study. The frequency is nearly linearly swept during *T*_l_ around the HS2 pulse center ($-T_{l}/2\leq t\leq T_{l}/2$, center row; shaded area). The linear frequency sweep well‐approximates the HS2 pulse phase during *T*_l_ (bottom row). (B) Quadratic phase in the HS2 excitation profile. Bloch simulation was computed using the Cayley‐Klein parameters (*α*, *β*)^T^. The quadratic phase in the HS2 excitation profile (blue) matched well with the one from the linear sweep approximation (red; Equation (4)). The quadratic phase was mostly attributed to the *β* parameter (green; *φ*(*Δω*_0_) ≈ arg(*β*(*Δω*_0_))); the phase profile of the *α* parameter was nearly flat (purple).

FM pulses with linear (or near linear) frequency sweeps such as chirp and hyperbolic secant (HS) pulses are well‐known to generate quadratic phases in the excitation profile [11]. With linearly frequency‐swept pulses, spin isochromats are excited sequentially in space at the time of on‐resonance, $t_{e}$, as a tipping around the RF/B_1_^+^ field and then freely precess after excitation [12, 13]. Thus, the quadratic phase generated by the frequency‐swept pulse, $\varphi \left(∆\omega_{0}\right)$, can be approximated as

(1) $$\varphi \left(∆\omega_{0}\right)=\varphi_{\mathrm{FM}}\left(t_{e}\left(∆\omega_{0}\right)\right)+2\pi \int_{t_{e}}^{0}∆\omega_{0}\mathrm{dt},$$

where $∆\omega_{0}$ is off‐resonance frequency and $\varphi_{\mathrm{FM}}\left(t_{e}\right)$ is the FM pulse phase at the time of excitation, $t_{e}\left(∆\omega_{0}\right)$, for the isochromat having resonance offset $∆\omega_{0}$. For a period of time $T_{l}$ about the center of FM pulses ($T_{l}$≤ pulse width, $T_{p}$), the frequency sweep can be approximated to be linear (Figure 1A):

(2) $$\omega_{\mathrm{FM}}\left(t\right)\approx -\frac{\mathrm{bw}}{T_{l}}t,t\in \left[-\frac{T_{l}}{2},\frac{T_{l}}{2}\right],$$

$$t_{e}\left(∆\omega_{0}\right)\approx -\frac{T_{l}}{\mathrm{bw}}∆\omega_{0},$$

where bw is the bandwidth of the FM pulse. Therefore, Equation (1) is expressed using $\varphi_{\mathrm{FM}}\left(t\right)=2\pi \int \omega_{\mathrm{FM}}\left(t^{\prime }\right){\mathrm{dt}}^{\prime }$:

(3) $$\varphi \left(∆\omega_{0}\right)\approx \pi T_{l}\mathrm{bw}{\left(\frac{∆\omega_{0}}{\mathrm{bw}}\right)}^{2},$$

in which $∆\omega_{0}/\mathrm{bw}$ is the resonance offset normalized by the FM pulse bandwidth. The linear sweep duration $T_{l}$ is unique to individual FM pulses (e.g., $T_{l}=T_{p}$ for chirp pulses) and is proportional to $T_{p}:$ $T_{l}=r\cdot T_{p}\left(r\leq 1\right)$. Using the linear sweep time fraction *r*, Equation (3) can be represented with time bandwidth product of the FM pulse, *TBP* (= *T*_*p*_⋅*bw*):

(4) $$\varphi \left(∆\omega_{0}\right)\approx \pi r\cdot \mathrm{TBP}{\left(\frac{∆\omega_{0}}{\mathrm{bw}}\right)}^{2}$$

Therefore, the quadratic phase in the profile is determined by *r* and *TBP* of the FM pulse.

This quadratic phase obtained under the linear sweep approximation matches well with the phase profile predicted by Bloch simulation (Figure 1B). In this study, we computed Bloch simulation in the spin domain (i.e., spinor representation) using the Cayley‐Klein parameters [14, 15], (*α*, *β*)^T^, for convenience to distinguish different dephased spin states (i.e., to differentiate spin‐ and stimulated‐echo components) in the next section. Moreover, while the phase of *α* was nearly flat, especially around the center of the profile, the phase of *β* nearly perfectly explained the quadratic phase in *M*_*xy*_ (and that from the linear sweep approximation); thereby, the quadratic phase in the FM pulse profile is mainly attributed to *β* (i.e., arg(*β*(*Δω*_0_)) ≈ *φ*(*Δω*_0_)). In this article, we exemplified the linear sweep approximation using flattened HS pulses (specifically, HS2 pulses) [11], but the approximation is also valid for other types of FM pulses with a linear frequency sweep such as chirp and WURST [16] pulses.

### 2.2 Consistent Quadratic Phase in the Fast Spin Echo With FM Pulses

To investigate the condition to generate a consistent phase in all echoes in an echo train in FM‐FSE, we consider a simple FSE sequence with two refocused echoes *s*_1,2_ (i.e., one excitation FM pulse (RF_exc_) and two refocus FM pulses (RF_ref1,2_) with an echo spacing *τ*): RF_exc_—*τ*/2—RF_ref1_—*τ*/2—*s*_1_– *τ*/2—RF_ref2_—*τ*/2—*s*_2_. For simplicity, the three FM pulses are assumed to have a common linear sweep time fraction, *r*. Common pulse parameters (e.g., pulse truncation level) are used in the design of the three FM pulses. During the FSE sequence, a constant magnetic field offset in time, *Δ*B_0_, is applied to generate an off‐resonance distribution. The Cayley‐Klein parameters for RF_exc_, RF_ref1_, and RF_ref2_ are represented by ($\alpha_{e}$, $\beta_{e}$)^T^, ($\alpha_{r1}$, $\beta_{r1}$)^T^, and ($\alpha_{r2}$, $\beta_{r2}$)^T^, respectively. Using these, the first refocused echo at *s*_1_, ($\alpha_{s1}$, $\beta_{s1}$)^T^, is given by [15, 17]:

(5) $$\begin{array}{ll} \left(\begin{array}{c} \alpha_{s1}\\ \beta_{s1} \end{array}\right) & =\left(\begin{array}{cc} \varepsilon^{-\frac{1}{2}} & 0\\ 0 & \varepsilon^{\frac{1}{2}} \end{array}\right)\left(\begin{array}{cc} \alpha_{r1} & {-\beta }_{r1}^{*}\\ \beta_{r1} & \alpha_{r1}^{*} \end{array}\right)\left(\begin{array}{cc} \varepsilon^{-\frac{1}{2}} & 0\\ 0 & \varepsilon^{\frac{1}{2}} \end{array}\right)\left(\begin{array}{cc} \alpha_{e} & {-\beta }_{e}^{*}\\ \beta_{e} & \alpha_{e}^{*} \end{array}\right)\left(\begin{array}{c} 1\\ 0 \end{array}\right)\\ & =\left(\begin{array}{c} \varepsilon^{-1}\alpha_{e}\alpha_{r1}-\beta_{e}\beta_{r1}^{*}\\ \alpha_{e}\beta_{r1}+\varepsilon \beta_{e}\alpha_{r1}^{*} \end{array}\right), \end{array}$$

where

$$\varepsilon =e^{\pi i∆\omega_{0}\tau }$$

is the amount of spin phase accumulated during the *τ*/2 period due to the constant offset *Δω*_0_ = *γΔ*B_0_ (*γ* is the gyromagnetic ratio). Then, the transverse magnetization at *s*_1_ is given by:

(6) $$\begin{array}{cc} {\left.M_{\mathrm{xy}}\right|}_{s1} & =2\alpha_{s1}^{*}\beta_{s1}=2\varepsilon^{2}\alpha_{e}^{*}\beta_{e}{\left(\alpha_{r1}^{*}\right)}^{2}+2\varepsilon \left(\alpha_{e}\alpha_{e}^{*}-\beta_{e}\beta_{e}^{*}\right)\alpha_{r1}^{*}\beta_{r1}\\ & \;-2\alpha_{e}\beta_{e}^{*}{\left(\beta_{r1}\right)}^{2}. \end{array}$$

Assuming sufficient dephasing during the *τ*/2 period (i.e., ignoring the terms with $\varepsilon^{m}\;\left(|m|>0\right)$), only the last term ($-2\alpha_{e}\beta_{e}^{*}{\left(\beta_{r1}\right)}^{2}$) contributes to the echo signal at *s*_1_.

Similarly, the second refocused echo at *s*_2_, ($\alpha_{s2}$, $\beta_{s2}$)^T^, and its transverse magnetization are given by:


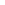

(7) $$\begin{array}{ll} \left(\begin{array}{c} \alpha_{s2}\\ \beta_{s2} \end{array}\right) & =\left(\begin{array}{cc} \varepsilon^{-\frac{1}{2}} & 0\\ 0 & \varepsilon^{\frac{1}{2}} \end{array}\right)\left(\begin{array}{cc} \alpha_{r2} & {-\beta }_{r2}^{*}\\ \beta_{r2} & \alpha_{r2}^{*} \end{array}\right)\left(\begin{array}{cc} \varepsilon^{-\frac{1}{2}} & 0\\ 0 & \varepsilon^{\frac{1}{2}} \end{array}\right)\left(\begin{array}{c} \alpha_{s1}\\ \beta_{s1} \end{array}\right)\\ & =\left(\begin{array}{c} \varepsilon^{-1}\alpha_{s1}\alpha_{r2}-\beta_{s1}\beta_{r2}^{*}\\ \alpha_{s1}\beta_{r2}+\varepsilon \beta_{s1}\alpha_{r2}^{*} \end{array}\right), \end{array}$$

(8) $$\begin{array}{cc} {\left.M_{\mathrm{xy}}\right|}_{s2} & =2\alpha_{s2}^{*}\beta_{s2}\\ & =\varepsilon^{4}\mu_{e}{\left(\alpha_{r1}^{*}\right)}^{2}{\left(\alpha_{r2}^{*}\right)}^{2}+\varepsilon^{3}\nu_{e}\mu_{r1}{\left(\alpha_{r2}^{*}\right)}^{2}\\ & \;-\varepsilon^{2}\left(\mu_{e}\alpha_{r1}^{*}\beta_{r1}^{*}\mu_{r2}+\mu_{e}^{*}{\left(\beta_{r1}\alpha_{r2}^{*}\right)}^{2}\right)+\varepsilon \nu_{e}\nu_{r1}\mu_{r2}\\ & \;-\mu_{e}^{*}\alpha_{r1}\beta_{r1}\mu_{r2}+\mu_{e}{\left(\beta_{r1}^{*}\right)}^{2}{\left(\beta_{r2}\right)}^{2}-\varepsilon^{-1}\nu_{e}\mu_{r1}^{*}{\left(\beta_{r2}\right)}^{2}\\ & \;-\varepsilon^{-2}\mu_{e}^{*}{\left(\alpha_{r1}\right)}^{2}{\left(\beta_{r2}\right)}^{2}, \end{array}$$

where

(9) $$\mu_{\mathrm{xx}}=2\alpha_{\mathrm{xx}}^{*}\beta_{\mathrm{xx}}$$

and

(10) $$\upsilon_{\mathrm{xx}}=\alpha_{\mathrm{xx}}\alpha_{\mathrm{xx}}^{*}-\beta_{\mathrm{xx}}\beta_{\mathrm{xx}}^{*}$$

with *xx* representing *e*, *r1*, or *r2*. When the dephasing is sufficiently strong, only two terms, $-\mu_{e}^{*}\alpha_{r1}\beta_{r1}\mu_{r2}$ and $\mu_{e}{\left(\beta_{r1}^{*}\right)}^{2}{\left(\beta_{r2}\right)}^{2},$ contribute to the second echo signal *s*_2_. The first term is an odd refocused/parity echo and the second one is an even refocused/parity echo, which corresponds to the stimulated‐echo and double spin‐echo component, respectively.

The most common spin‐echo sequence using FM pulses is the adiabatic double spin‐echo sequence which uses 90° excitation with a flat magnetization phase profile (e.g., non‐selective excitation) and a pair of adiabatic full‐passage (AFP) pulses [6]; thereby, $\alpha_{e}=\beta_{e}=\sqrt{2}/2$,$\;\alpha_{r1}=\alpha_{r2}$, and $\beta_{r1}=\beta_{r2}$. In this case, the phase profile of the spin‐echo signal in *s*_1_ (Equation 6) is given with the linear sweep approximation (Equation 4) by:

(11) $$\arg\left(-2\alpha_{e}\beta_{e}^{*}{\left(\beta_{r1}\right)}^{2}\right)=\arg\left(-{\left(\beta_{r1}\right)}^{2}\right)\approx 2\pi r\cdot \mathrm{TBP}{\left(\frac{∆\omega_{0}}{\mathrm{bw}}\right)}^{2}+\pi .$$

Similarly, the phase of the stimulated‐ and double spin‐echo signals in *s*_2_ (Equation 8) is given by:

(12) $$\arg\left(-\mu_{e}^{*}\alpha_{r1}\beta_{r1}\mu_{r2}\right)\approx \arg\left(-{\left(\beta_{r1}\right)}^{2}\right)\approx 2\pi r\cdot \mathrm{TBP}{\left(\frac{∆\omega_{0}}{\mathrm{bw}}\right)}^{2}+\pi ,$$

and

(13) $$\arg\left(\mu_{e}{\left(\beta_{r1}^{*}\right)}^{2}{\left(\beta_{r2}\right)}^{2}\right)=\arg\left({\left({\beta_{r1}\beta }_{r1}^{*}\right)}^{2}\right)=0.$$

The phase of the double spin‐echo is perfectly refocused independently of *α*; therefore, the double spin‐echo component always yields flat phase regardless of the RF pulse type [18]. The quadratic phase of the stimulated‐echo (Equation 12) is consistent with that of the spin‐echo in *s*_1_ (Equation 11), since these two are both odd parity/refocused echoes [19]. The two refocusing FM pulses in the double spin‐echo sequence needs to be adiabatic (i.e., AFP pulses); otherwise, the second echo *s*_2_ will suffer interference of the stimulated and double spin echoes due to their inconsistent phase. When the AFP pulse is repeated to generate s_*n*_ (*n* ≥ 3), odd echoes (*s*_3_, *s*_5_, …) show the consistent quadratic phase (Equations 11 and 12), and even echoes (*s*_4_, *s*_6_, …) are composed of even parity/refocused echoes with flat phase (Equation 13). Then, only even or odd echoes are typically used in FSE with AFP refocusing pulses (“FID” and “spectral” echoes) [1, 2, 10]. In the FSE sequence herein, the excitation and refocusing RF pulses have a common RF phase (i.e., CP phase [20]); as such, there is an extra π phase shift between even and odd parity echoes in addition to the quadratic phase (Equations (11), (12), (13)).

Another spin echo‐based technique using FM pulses is the matched RF spin‐echo sequence [7, 8, 17], where the quadratic phase generated by an excitation FM pulse is compensated by its matched refocusing FM pulse. The phase profile generated by RF_exc_ and that of the first echo *s*_1_ are represented by using the time bandwidth product of the excitation and the first refocusing FM pulses, *TBP*_*e*_ and *TBP*_*r1*_:

(14) $$\arg\left(2\alpha_{e}^{*}\beta_{e}\right)\approx \arg\left(\beta_{e}\right)\approx \pi r\cdot {\mathrm{TBP}}_{e}{\left(\frac{∆\omega_{0}}{\mathrm{bw}}\right)}^{2},$$

and

(15) $$\begin{array}{cc} \arg\left(-2\alpha_{e}\beta_{e}^{*}{\left(\beta_{r1}\right)}^{2}\right) & \approx \arg\left(-\beta_{e}^{*}{\left(\beta_{r1}\right)}^{2}\right)\\ & \approx \pi r\left(2{\mathrm{TBP}}_{r1}-{\mathrm{TBP}}_{e}\right)\cdot {\left(\frac{∆\omega_{0}}{\mathrm{bw}}\right)}^{2}+\pi . \end{array}$$

Therefore, the quadratic phase of the spin‐echo *s*_1_ can be removed by setting the time bandwidth product to satisfy *TBP*_*e*_ = 2*TBP*_*r1*_.

When the second refocusing RF pulse (RF_ref2_ with *TBP*_*r*2_) is applied after the spin‐echo *s*_1_, the phase profile of the stimulated‐ and double spin‐echo components in the second echo *s*_2_ (Equation 8) is given using the time bandwidth products of the excitation and the two refocusing FM pulses by:

(16) $$\begin{array}{cc} \arg\left(-\mu_{e}^{*}\alpha_{r1}\beta_{r1}\mu_{r2}\right) & \approx \arg\left(-\beta_{e}^{*}\beta_{r1}\beta_{r2}\right)\\ & \approx \pi r\left({\mathrm{TBP}}_{r1}+{\mathrm{TBP}}_{r2}-{\mathrm{TBP}}_{e}\right)\cdot {\left(\frac{∆\omega_{0}}{\mathrm{bw}}\right)}^{2}+\pi , \end{array}$$

and

(17) $$\begin{array}{cc} \arg\left(\mu_{e}{\left(\beta_{r1}^{*}\right)}^{2}{\left(\beta_{r2}\right)}^{2}\right) & \approx \arg\left(\beta_{e}{\left(\beta_{r1}^{*}\right)}^{2}{\left(\beta_{r2}\right)}^{2}\right)\\ & \approx \pi r\left({\mathrm{TBP}}_{e}+2{\mathrm{TBP}}_{r2}-2{\mathrm{TBP}}_{r1}\right)\cdot {\left(\frac{∆\omega_{0}}{\mathrm{bw}}\right)}^{2}. \end{array}$$

In this article, we formulate a condition where all refocused echoes show a consistent quadratic phase. In such a condition, the quadratic phase given by Equations ((14), (15), (16), (17)) needs to be consistent; as such the FM pulses must satisfy:

(18) $${\mathrm{TBP}}_{e}={\mathrm{TBP}}_{r1}={\mathrm{TBP}}_{r2}=\mathrm{TBP}.$$

When this condition is satisfied, all the dephased states in Equations (6) and (8) (the terms with $\varepsilon^{m},|m|>0$) also show the consistent quadratic phase. Accordingly, when one continues to apply FM pulses with the common time bandwidth product (*TBP*) to refocus an FSE echo train s_*n*_ (*n* ≥ 3), all echoes show the consistent quadratic phase.

### 2.3 Flip Angle Nonlinearity and B_1_ ^+^‐Dependent Phase in FM Pulses

**FIGURE 2:**
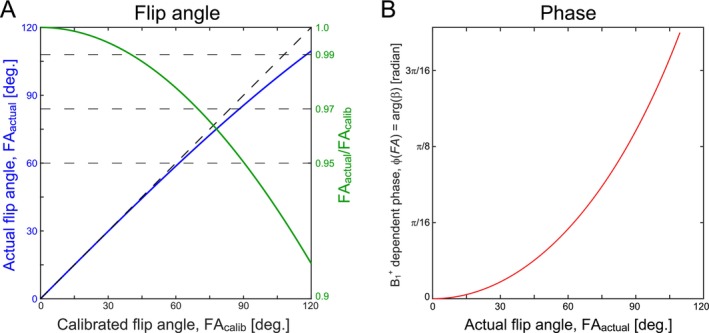
Flip angle nonlinearity (A) and B_1_^+^‐dependent phase (B) of the flattened hyperbolic secant (HS2) pulse used in this study. The flip angle, FA_actual_, is linear to the calibrated flip angle, FA_calib_, for low flip angles, but it is underestimated as the flip angle increases (A; blue, left axis); the error is less than 1% up to around 40° and it goes up to 2.4% and 5.9% for 60° and 90°, respectively (green, right axis). While a common quadratic phase is observed regardless of the flip angle, there is a constant phase offset depending on the flip angle (Figure S1); the flip angle/B_1_^+^‐dependent phase quadratically increases along with an increase of the flip angle (B).

RF transmitter power is typically calibrated based on the integral of the RF pulse table, $\left|\int B_{1}\left(t\right)\mathrm{dt}\right|$, and the reference voltage. For amplitude‐modulated (AM) pulses such as hard, sinc and Gaussian pulses, the transmitter power calibrated using the pulse integral is perfectly accurate for the center isochromat regardless of the flip angle: flip angle = $c\left|\int B_{1}\left(t\right)\mathrm{dt}\right|$, where *c* is a constant scaling factor calculated based on the reference voltage that is typically calibrated using a hard pulse with $\left|\int B_{1}\left(t\right)\mathrm{dt}\right|=1$. With FM pulses, this linear relation works only under the low tip angle approximation [21], even for the center isochromat. Therefore, the flip angle of FM pulses starts showing nonlinearity as the flip angle increases [7] (Figure 2A). For the HS2 pulse used herein, the actual flip angle achieved is slightly less than the flip angle calibrated with the linear relation; the difference is less than 1% up to 40° and it goes up to 2.4% and 5.9% for 60° and 90°, respectively.

FM pulses are also known to introduce B_1_^+^‐dependent phase in the spin magnetization. The B_1_^+^‐dependent phase produced by FM pulses was used previously for B_1_ mapping with the adiabatic double spin‐echo sequence [22] and the matched RF spin‐echo sequence [23]. More recently, the B_1_^+^‐dependent phase that's produced by FM pulses was exploited to enable spatial encoding with an RF coil having a monotonically changing B_1_^+^ in space [24]. The B_1_^+^‐dependent phase, which is the same as the flip‐angle‐dependent phase *ϕ*(*FA*), was determined for the center isochromat (i.e., at the vertex of the quadratic phase). As such, $\phi \left(\mathrm{FA}\right)=\arg\left(M_{\mathrm{xy}}\right)=\arg\left(\beta \right)$ (Figure 2B), because arg(α)=0 for the center isochromat regardless of the flip angle (Figure S1).

## 3 Methods

All MRI scans were performed with a clinical 3 T MRI scanner (Prisma, Siemens Healthineers, Erlangen, Germany) with a body transmit coil and a 32‐channel head receive coil under an Internal Review Board approved protocol. Study participants recruited from the local volunteer pool (*N* = 10) provided written informed consent prior to participation.

### 3.1 3D FM‐FSE Sequence With Consistent Quadratic Phase

**FIGURE 3:**
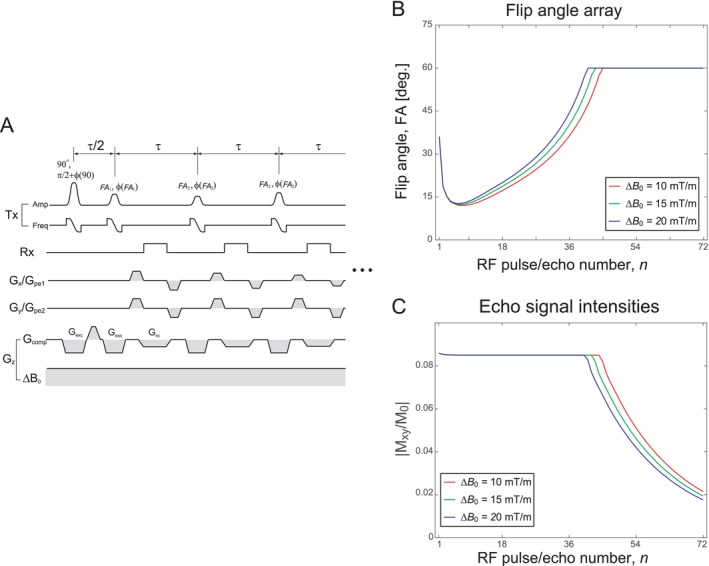
(A) Sequence diagram of the 3D fast spin‐echo (FSE) sequence with frequency‐modulated RF pulses. Variable flip angles were used in the refocusing RF pulses to achieve flat echo signals in FSE (B). The flip angle array for the refocusing pulses was optimized for three inhomogeneous field gradients of 10/15/20 mT/m using *T*_1_/*T*_2_ and ADC values for the brain at 3 T (*T*_1_/*T*_2_ = 1000/100 ms and ADC = 1.0 × 10^−3^ mm^2^/s). The echo signals were nearly flat until the refocusing flip angle reached the maximum of 60° (C). The refocusing flip angles were higher under higher inhomogeneous field gradients to compensate for the signal attenuation due to diffusion.

The consistent quadratic phase condition was implemented in 3D FSE with FM pulses by employing excitation and refocusing FM pulses that satisfy the forementioned condition (Equation 18) (Figure 3A). A HS2 pulse with 12‐kHz bandwidth and 3.6‐ms pulse width (*TBP* = 43.2) was used for both excitation and refocusing RF pulses. The CPMG phase condition (π/2 and 0 phase for excitation and refocus, respectively) was used to avoid the interference of even and odd refocused echo (π phase shift observed with the CP phase condition above), for example, Equations ((11), (12), (13)). The different B_1_^+^‐dependent phases produced by HS2 excitation and refocusing (Figure 2B) were compensated by adjusting the RF pulse phase on top of the CPMG phase.

Refocusing flip angles in 3D FM‐FSE were determined with prospective EPG [25] to achieve flat echo signals in refocused echoes (i.e., to avoid degradation of the point spread function). Since we assume relatively large magnetic field inhomogeneity, diffusion effects were also included in the prospective EPG [26, 27, 28]; Similarly to the previous work [25], variable refocusing flip angles were calculated to make the target signal at echo *n* ($S_{\text{target}}\left(n\right)$) asymptotically reaching $S_{\text{target}}$: $S_{\text{target}}\left(1\right)=1.02\cdot S_{\text{target}}$ and $S_{\text{target}}\left(n\right)=\left(S_{\text{target}}\left(n-1\right)+S_{\text{target}}\right)/2$(*n* > 1). In the prospective EPG, *T*_1_/*T*_2_ values and apparent diffusion coefficient (ADC) were set to the values for in vivo human brain in literature [29, 30] (*T*_1_/*T*_2_ = 1000/100 ms and ADC = 1.0 × 10^−3^ mm^2^/s). In this study, the flip angle array was calculated assuming three inhomogeneous field gradients *ΔB*_0_ = 10/15/20 mT/m. The flip angles were slightly higher with higher *ΔB*_0_ to compensate for the signal attenuation due to diffusion. With the calculated flip angle array, the echo signals were nearly flat until the flip angle reached a maximum of 60° (Figure 3B,C). The maximum flip angle of 60° was empirically determined based on the SAR limit for in vivo brain imaging at 3 T. Therefore, the excitation HS2 pulse had the highest flip angle of 90°. The calculated flip angle array was adjusted to compensate for the nonlinearity of the HS2 pulse flip angle by applying the flip angle scaling factor (Figure 2A).

### 3.2 Extended Phase Graph (EPG) Simulation

EPG simulations were performed to validate the consistent quadratic phase in the refocused echoes in 3D FM‐FSE. To test the impacts from the B_1_^+^‐dependent phase, EPG simulation was computed with and without the B_1_^+^‐dependent phase adjustment. RF pulse phase, *θ*, can be incorporated in the Cayley‐Klein parameter [31]:

(19) $$\left(\begin{array}{l} \alpha_{\theta }\\ \beta_{\theta } \end{array}\right)=\left(\begin{array}{ll} e^{-i\frac{\theta }{2}} & 0\\ 0 & e^{i\frac{\theta }{2}} \end{array}\right)\left(\begin{array}{ll} \alpha & -\beta^{*}\\ \beta & \alpha^{*} \end{array}\right)\left(\begin{array}{ll} e^{i\frac{\theta }{2}} & 0\\ 0 & e^{-i\frac{\theta }{2}} \end{array}\right)\left(\begin{array}{l} 1\\ 0 \end{array}\right)=\left(\begin{array}{l} \alpha \\ e^{i\theta }\beta \end{array}\right)$$

Therefore, to compensate for the B_1_^+^/*FA*‐dependent phase *ϕ*(*FA*) (= arg(*β*)) at the center of the profile (Figure 2B), the RF phase *θ* needs to be set to:

(20) θ=−ϕ(FA).

The Cayley‐Klein parameter (*α, β*)^T^ was converted to a 3 × 3 RF matrix for EPG simulation [31]:

$${\left(\begin{array}{l} F\\ F^{*}\\ Z \end{array}\right)}^{+}=\left(\begin{array}{lll} {\left({\alpha_{\theta }}^{*}\right)}^{2} & -{\beta_{\theta }}^{2} & 2\alpha_{\theta }^{*}\beta_{\theta }\\ -\;{\left(\beta_{\theta }^{*}\right)}^{2} & {\alpha_{\theta }}^{2} & 2\alpha_{\theta }\beta_{\theta }^{*}\\ -\;\alpha_{\theta }^{*}\beta_{\theta }^{*} & -\alpha_{\theta }\beta_{\theta } & \alpha_{\theta }\alpha_{\theta }^{*}-\beta_{\theta }\beta_{\theta }^{*} \end{array}\right)\;{\left(\begin{array}{l} F\\ F^{*}\\ Z \end{array}\right)}^{-}$$

(21) $$=\left(\begin{array}{lll} {\left(\alpha^{*}\right)}^{2} & -e^{2i\theta }\beta^{2} & 2e^{i\theta }\alpha^{*}\beta \\ -\;e^{-2i\theta }{\left(\beta^{*}\right)}^{2} & \alpha^{2} & 2e^{-i\theta }\alpha \beta^{*}\\ -\;e^{-i\theta }\alpha^{*}\beta^{*} & -e^{i\theta }\alpha \beta & \alpha \alpha^{*}-\beta \beta^{*} \end{array}\right)\;{\left(\begin{array}{l} F\\ F^{*}\\ Z \end{array}\right)}^{-}$$

Using this matrix, EPG simulation was computed incorporating *T*_1_/*T*_2_ relaxation and diffusion [26]. In EPG simulation, *T*_1_/*T*_2_/ADC values were set to 1000 ms/100 ms/1.0 × 10^−3^ mm^2^/s for brain tissues [29, 30] and 4000 ms/1700 ms/3.0 × 10^−3^ mm^2^/s for cerebrospinal fluid (CSF) [29, 32, 33], respectively.

### 3.3 Relaxation Effects

While the maximum flip angle in clinical 3D FSE scans is typically set to ≥ 120°, the maximum flip angle of the FM refocusing pulses in this study (60°) is significantly lower because of the severe SAR limitation associated with the broad bandwidth of the FM pulses. When refocusing flip angles in FSE are less than 180°, the signal decay of the refocused echo train is a mixture of *T*_1_ and *T*_2_ relaxation depending on the time fractions of longitudinal and transverse magnetization states in the magnetization pathways [34, 35]; low flip angle refocusing increases the contribution of *T*_1_ relaxation. Therefore, with low refocusing flip angles, a long‐lasting echo train is available for imaging, because *T*_1_ relaxation is typically much slower than *T*_2_ relaxation. The FSE echo signals in the EPG simulation, $S_{\mathrm{EPG}}\left(T_{1},T_{2},\mathrm{ADC}\right)$, at TE=n·τ(n=1,2,3…) can be modeled using *T*_2_ decay time fraction *f*_*t*_ as [34, 35]:

(22) $$\begin{array}{cc} S_{\mathrm{EPG}}\left(T_{1},T_{2},\mathrm{ADC}\right) & =S_{\mathrm{EPG}}\left(T_{1}=\infty ,T_{2}=\infty ,\mathrm{ADC}\right)\\ & \;\cdot \exp\left\{-\left(\frac{f_{t}}{T_{2}}+\frac{1-f_{t}}{T_{1}}\right)\mathrm{TE}\right\}. \end{array}$$

Then, *f*_*t*_ is computed from the ratio of the two EPG simulation results, $R_{\text{relax}}$, by:

(23) $$f_{t}=-\left(\log R_{\text{relax}}+\frac{\mathrm{TE}}{T_{1}}\right)\cdot \frac{T_{1}T_{2}}{T_{1}-T_{2}}\cdot \frac{1}{\mathrm{TE}},$$

where

(24) $$\;R_{\text{relax}}=\frac{S_{\mathrm{EPG}}\left(T_{1},T_{2},\mathrm{ADC}\right)}{S_{\mathrm{EPG}}\left(T_{1}=\infty ,T_{2}=\infty ,\mathrm{ADC}\right)}.$$

Based on *f*_*t*_, effective TE (*TE*_*eff*_) is defined in two ways, as done in previous studies [25, 35]:

(25) $${\mathrm{TE}}_{\mathrm{eff}}=f_{t}\cdot \mathrm{TE},$$

(26) $$TE_{\mathrm{eff}}^{\prime }=-T_{2}\cdot \log R_{\text{relax}}=\left\{f_{t}+\left(1-f_{t}\right)\frac{T_{2}}{T_{1}}\right\}\cdot \mathrm{TE}=\bar{f_{t}}\cdot \mathrm{TE}.$$

While *TE*_*eff*_ counts only for *T*_2_ relaxation in the signal decay, *TE'*_*eff*_ includes *T*_1_ relaxation as well (i.e., the weighted average of *T*_1_ and *T*_2_ relaxation, $\bar{f_{t}}$). Accordingly, as mentioned previously [25], ${\mathrm{TE}}_{\mathrm{eff}}^{\prime }={\mathrm{TE}}_{\mathrm{eff}}$ when ignoring *T*_1_ relaxation (i.e., $T_{1}=\infty$), which is reasonable because of *T*_2_ < < *T*_1_ for most soft tissues. In this study, the *T*_2_ decay time fraction and effective TE were calculated for brain tissues at 3 T (*T*_1_/*T*_2_ = 1000/100 ms and ADC = 1.0 × 10^−3^ mm^2^/s) to evaluate *T*_2_ contrasts with 3D FM‐FSE. The echo signal decay also includes signal attenuation associated with diffusion, but it was not explicitly incorporated in the model herein.

### 3.4 MRI Experiments

In this study, since the static magnetic field in the clinical MRI scanner is highly homogeneous, a linear magnetic field inhomogeneity was introduced by applying a linear gradient field during the entire 3D FM‐FSE scans using the z channel of the linear gradient coil set. Experiments were performed with the linear field inhomogeneity *ΔB*_0_ set to three different values, 10/15/20 mT/m, which correspond to 61/92/123 kHz resonance variation over a 14.4‐cm field of view (FOV). The linear field inhomogeneity was partly compensated during the RF pulses and signal detection to reduce bandwidth (12 kHz and 40 kHz during RF pulses and signal readout, respectively) by modulating the z gradient amplitude (Figure 3A).

As noted above, our theoretical framework assumes complete dephasing of spins between all echoes, and thereby, terms with $\varepsilon^{m}\;\left(|m|>0\right)$ in Equation (6) can be ignored. As such, the gradient fields in the time periods between echoes, *τ*, must produce sufficiently large zeroth order gradient moment, *m*_0_:

(27) $$m_{0}=\int_{\left(n-1\right)\tau }^{n\tau }\left(G_{\text{comp}}\left(t\right)+∆B_{0}\right)\mathrm{dt}.$$

With the linear gradient field inhomogeneities used herein, *m*_0_ needs to satisfy $m_{0}\geq 2T_{\mathrm{acq}}\left(G_{\mathrm{ro}}+∆B_{0}\right)$ to generate ≥ 2π phase in each pixel (and to avoid interference of refocused and dephased state signals), where $T_{\mathrm{acq}}$ is the acquisition time in each readout. In the current experimental setting, $m_{0}/T_{\mathrm{acq}}\left(G_{\mathrm{ro}}+∆B_{0}\right)$ was 4.8/6.1/7.4 for the linear inhomogeneous gradient field of 10/15/20 mT/m, respectively, and as such sufficient dephasing was achieved.

The flip angle array obtained with the prospective EPG method was used in both *T*_1_‐ and *T*_2_‐weighted imaging in in vivo brain imaging with different *k*‐space filling orders in the phase encoding plane (center‐out and sequential filling for *T*_1_‐ and *T*_2_‐weighted imaging, respectively). Sequence parameters were: TR = 900/1800 ms, TE = 22/396 ms, echo spacing (*τ*) = 11 ms, echo train length (etl) = 40/72, acquisition time (TA) = 4:30/5:00 for *T*_1_/*T*_2_‐weighted imaging, elliptical *k*‐space sampling and 1.5 mm isotropic spatial resolution.

For experimental validation of the proposed consistent quadratic phase formation, multi‐echo spin‐echo (MESE) acquisition was conducted for an agarose gel phantom with known relaxation and ADC values (*T*_1_/*T*_2_ = 1180/100 ms and ADC = 2.45 × 10^−3^ mm^2^/s). The *T*_1_, *T*_2_, and ADC values were experimentally measured with inversion recovery, spin‐echo, and Stejskal‐Tanner spin‐echo diffusion, respectively. B_1_^+^ mapping was also performed with the actual flip angle (AFI) method [36] to select an area with a flip angle matched with the ideal flip angle. The flip angle array of the refocusing pulses was optimized using these parameters for the phantom. Except for the spatial resolution in the phase‐encoding plane which was reduced to 4.5 mm to avoid a lengthy acquisition time in the MESE acquisition (TA = 19:30), other sequence parameters were the same as those in 3D FM‐FSE acquisition using the *T*_2_‐weighting specified above. To test the impacts from B_1_^+^ inhomogeneity in space, MESE acquisition was conducted with ±10% B_1_^+^ fields by adjusting the reference voltage, while the RF pulse phase was kept as optimal. The experimental results were compared with the EPG simulation for validation.

### 3.5 3D FM‐FSE in a Nonlinear Inhomogeneous Field

Performance of the developed 3D FM‐FSE sequence in a nonlinear inhomogeneous field was tested by introducing a nonlinear field inhomogeneity. The nonlinear inhomogeneous magnetic field was generated by adjusting the Z^2^ shim value in the B_0_‐shim coil set to its maximum, which resulted in approximately 3.5‐kHz off‐resonance over the agarose gel phantom used (∼23 cm along the Z dimension). B_0_ mapping was performed with gradient‐recalled echo acquisitions with two TEs (2.0 and 2.2 ms) to confirm the introduced off‐resonance distribution. The 3D FM‐FSE sequence parameters were identical to the brain scans for *T*_2_‐weighted imaging above, but the linear inhomogeneous field was turned off ($∆B_{0}$ = Z^2^ inhomogeneous field). To satisfy the sufficient dephasing for the dephased state magnetizations, crusher gradients were added before and after the readout gradient, $G_{\mathrm{ro}}$, in each *τ*; thereby, $m_{0}=4T_{\mathrm{acq}}G_{\mathrm{ro}}$, which is twice as high as the required gradient moment to ensure sufficient dephasing in the Z^2^ nonlinear inhomogeneous field. A conventional 3D FSE sequence that uses hard pulses for excitation and refocusing was also tested for comparison. The sequence parameters in the conventional 3D FSE were identical to 3D FM‐FSE, but the excitation and refocus RF pulses were replaced with 0.7‐ms hard pulses (*bw* ≈ 1.5 kHz) without the flip angle nonlinearity correction or the B_1_‐dependent phase adjustment; the 0.7‐ms pulse width is comparable to that in the clinical 3D FSE sequence at 3 T (e.g., SPACE). For comparison, the 3D FM‐FSE and conventional 3D FSE scans were performed without the inhomogeneous magnetic field to see their performance in homogeneous magnetic fields.

## 4 Results

### 4.1 EPG Simulation

**FIGURE 4:**
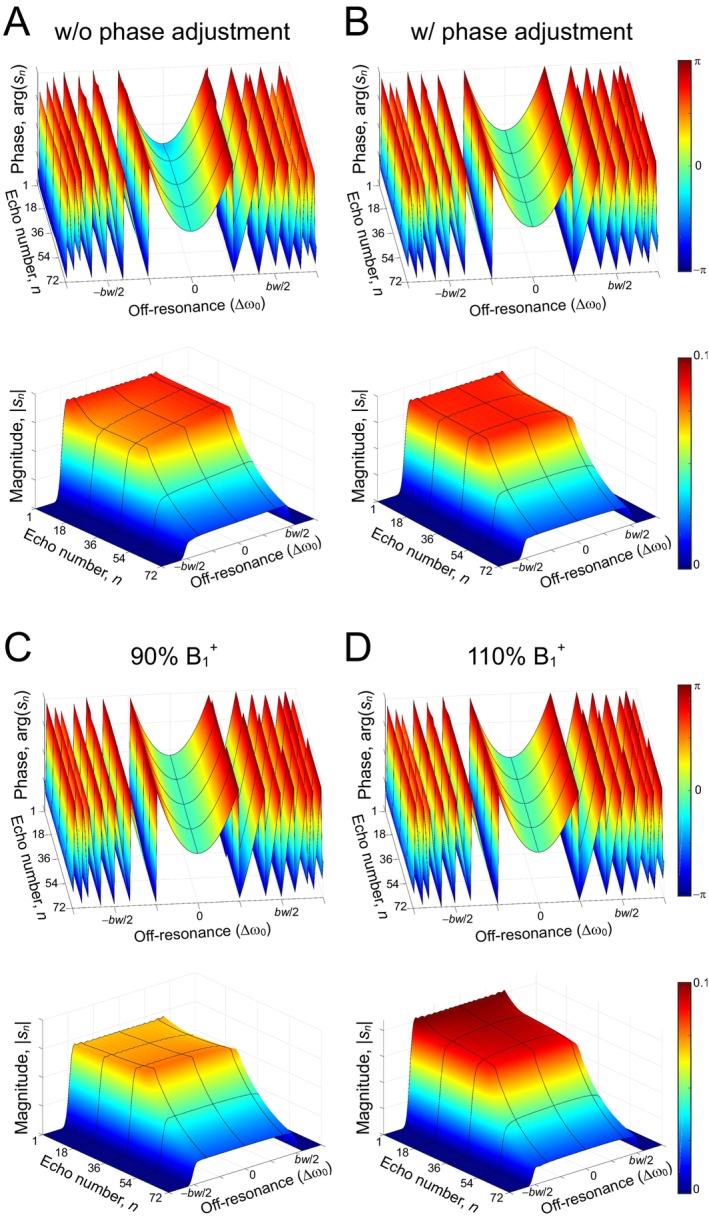
EPG simulation results of 3D FM‐FSE (A) without or (B) with the B_1_^+^‐dependent phase adjustment. Consistent quadratic phase is observed in all refocused echoes either with or without the B_1_^+^‐dependent phase adjustment (top). However, the magnitude profile produced without B_1_^+^‐dependent phase adjustment exhibits an increasing slant to the profile in echoes up to echo 18, whereas the magnitude profile was nearly flat in all echoes when using B_1_^+^‐dependent phase adjustment (bottom). EPG simulation was performed with 90% or 110% of the ideal B_1_^+^ to test impacts of the B_1_^+^ field mismatch between the ideal B_1_^+^ phase adjustment and the actual B_1_^+^ field (C,D). The quadratic phase is highly consistent in all echoes with the ±10% B_1_^+^ fields, which is comparable to the ideal case (B). The magnitude profile with the 90% or 110% B_1_^+^ shows a slight increase or decrease as the echo number increases until the flip angle reached the maximum, but the magnitude profile in each echo is nearly flat.

EPG simulation was performed with or without the B_1_^+^‐dependent phase adjustment for spin isochromats with the *T*_1_/*T*_2_ and ADC values for the agarose gel phantom. Consistent quadratic phase was observed either with or without the B_1_^+^‐dependent phase adjustment, but the first several echoes without the B_1_^+^‐dependent phase adjustment (*n* < 18) showed slightly more variation in the phase profile than those with the B_1_^+^‐dependent phase adjustment (Figure 4A,B). The magnitude profile showed more clear impacts between with and without the B_1_^+^‐dependent phase adjustment; the magnitude profile without the B_1_^+^‐dependent phase adjustment was nearly flat at echo 1, but it developed an increasing slant from one profile edge to the other until echo 18, and then the profile slant was consistent in the later echoes (∼14% drop from edge to edge). When the B_1_^+^‐dependent phase adjustment was used, the magnitude profile remained nearly flat in all echoes, although a minor intensity drop was observed from the profile center to the edges in the later echoes.

To see the impacts from B_1_^+^ inhomogeneity, EPG simulation was performed with 90% and 110% of the ideal B_1_^+^ magnitude while keeping the B_1_^+^‐dependent phase adjustment ideal (Figure 4C,D). Although the imposed B_1_^+^ scaling introduces mismatch between the actual B_1_^+^‐dependent phase and its ideal adjustment phase, the quadratic phase was similarly consistent with the ideal case (Figure 4B) for either 90% or 110% of the ideal B_1_^+^. The signal magnitude profile was nearly flat for each echo profile in both 90% and 110% cases, but the echo signal intensity slightly increased or decreased as the echo number increased for 90% or 110% until the flip angle reached the maximum at echo 44. Therefore, the B_1_^+^‐dependent phase adjustment is relatively resilient to B_1_^+^ inhomogeneity, at least, in a range of ±10% variation.

### 4.2 Relaxation Effects

**FIGURE 5:**
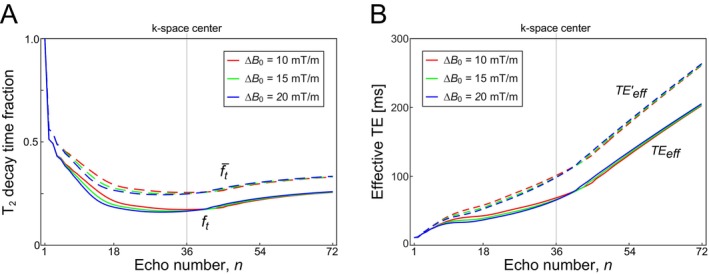
*T*_1_/*T*_2_ relaxation effects in the proposed 3D FM‐FSE with *T*_2_‐weighting. *T*_2_ relaxation time fraction, $f_{t}$, and its weighted average of *T*_1_ and *T*_2_ relaxation, $\bar{f_{t}}$, were calculated for three inhomogeneous field gradients of 10/15/20 mT/m (A; Equations ((23), (24), (25), (26))). *T*_2_ decay time fraction at the *k*‐space center echo in *T*_2_‐weighted imaging (echo 36) is comparable regardless of the inhomogeneous field gradient. Effective TE is ∼67 or ∼100 ms at the *k*‐space center echo (TE = 396 ms) either with $f_{t}$ or $\bar{f_{t}}$, which is reasonable to generate good *T*_2_‐contrasts in the brain at 3 T.

To evaluate *T*_2_ contrasts with the proposed 3D FM‐FSE sequence, *T*_2_ decay time fraction, $f_{t}$, and effective TE, *TE*_*eff*_, were calculated for three inhomogeneous field gradients (*ΔB*_0_ = 10, 15 and 20 mT/m; Figure 5). The impact of diffusion on $f_{t}$ was minor; $f_{t}$ was similar regardless of *ΔB*_0_. $f_{t}$ was relatively stable around 0.2 at echo 18 or later. At echo 36, where the *k*‐space center was sampled in *T*_2_‐weighted imaging, $f_{t}$ was relatively low (∼0.17); thereby, the effective TE was ∼67 and ∼100 ms with TE = 396 ms for ${\mathrm{TE}}_{\mathrm{eff}}$ and $\mathrm{TE}\prime_{\mathrm{eff}}$, respectively. These effective TE values are comparable to typical TE values used to achieve good *T*_2_ contrasts for brain tissues at 3 T (70–110 ms). With the experimental settings herein, *T*_1_ relaxation explained one third of the total signal decay associated with *T*_1_ and *T*_2_ relaxation because of the low $f_{t}$ at the *k*‐space center echo ($\frac{{\mathrm{TE}}_{\mathrm{eff}}}{TE_{\mathrm{eff}}^{\prime }}=\frac{f_{t}}{\bar{f_{t}}}∼0.67$).

### 4.3 Experimental Validation

**FIGURE 6:**
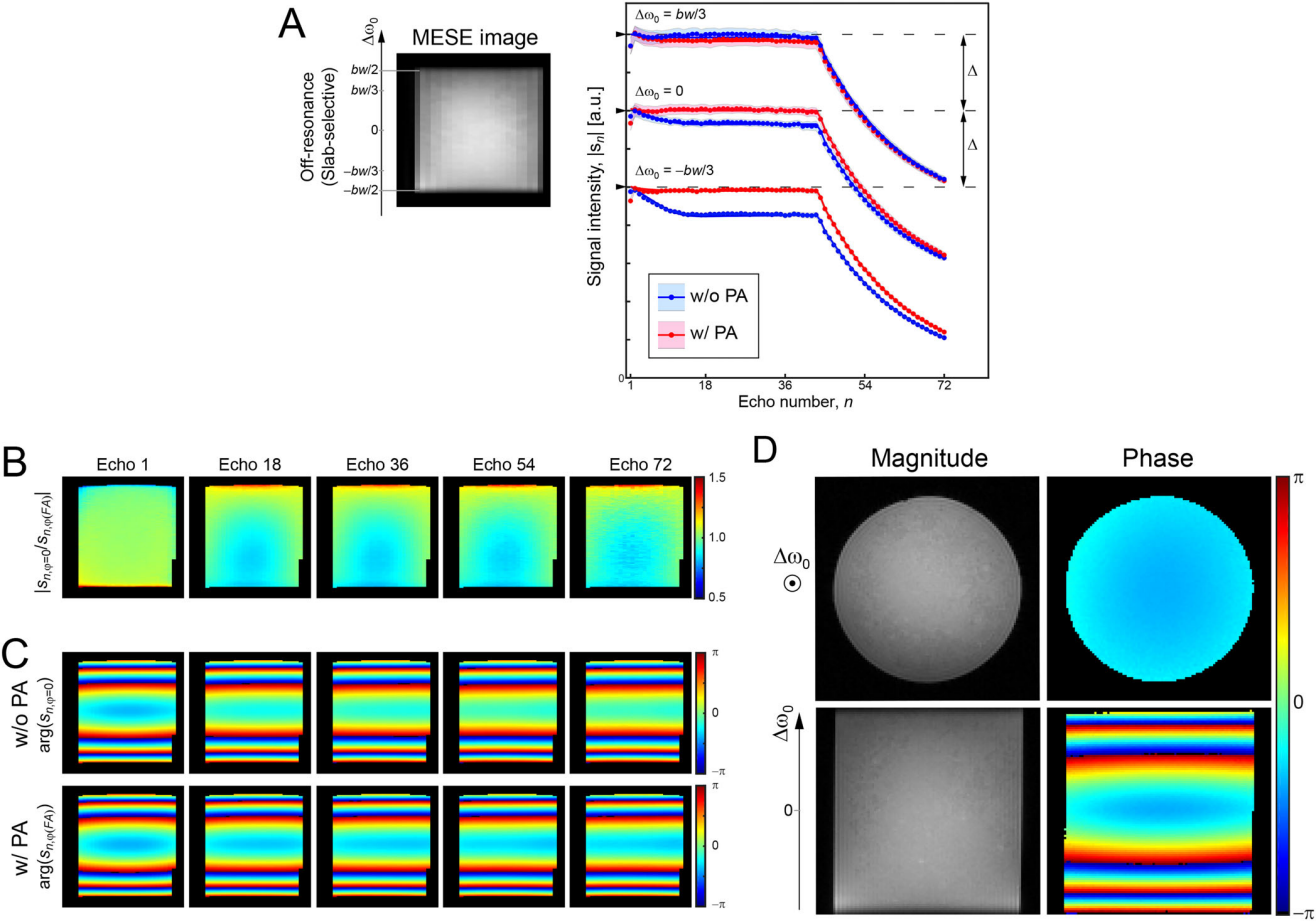
Experimental validation of the B_1_^+^‐dependent phase adjustment. Multi‐echo spin‐echo (MESE) acquisition was performed with and without the B_1_^+^‐dependent phase adjustment (w/PA and w/o PA) on an agarose gel phantom. (A) Without B_1_^+^‐dependent phase adjustment, the refocused echo signal at one edge of the profile gradually decreases as the echo number increases up to echo 18 while the other edge keeps constant signal intensity, which is consistent with the slanted profile in EPG simulation (Figure 4A). With B_1_^+^‐dependent phase adjustment, the profile is nearly flat for all echoes, but there are minor signal drops from the profile center to the edges at later echoes; $∆\omega_{0}=\pm \mathrm{bw}/3$ shows slightly lower signal intensities than the target signal in the prospective EPG (dashed lines in the plots). EPG simulation (solid lines) and experimental results (mean ± standard deviation; points and shaded area) were plotted with an offset *Δ* to avoid overlapping of the data. (B) Images of echoes 1, 18, 36, 54 and 72 show the effect of the slanted intensity profile. To remove the B_1_^−^ effects in the intensity profile, the intensity ratio of the two images with and without the B_1_^+^‐dependent phase adjustment was used. (C) Images of the quadratic phase in the off‐resonance (readout) dimension for echoes 1, 18, 36, 54 and 72. Consistent quadratic phase is observed in all echoes, but without B_1_^+^‐dependent phase adjustment the phase in echo 1 differs slightly from that in the later echoes (top row), whereas with B_1_^+^‐dependent phase adjustment the phase variation in echoes is noticeably reduced for all echoes (bottom row). (D) 3D FM‐FSE images of the agarose gel phantom. The quadratic phase does not need to be removed because the quadratic phase is consistent in all echoes. Therefore, the reconstructed image preserves the quadratic phase.

To validate the consistent quadratic phase generation and the impacts of the B_1_^+^‐dependent phase on the refocused echo profiles in 3D FM‐FSE, MESE measurements were conducted with an agarose gel phantom (Figure 6). MESE images acquired without B_1_^+^‐dependent phase adjustment reveal profiles that are increasingly slanted up to echo number 18, in agreement with the EPG simulation results (Figure 4A,B). Conversely, when using the B_1_^+^‐dependent phase adjustment, the profile remains nearly flat for all echoes, but similarly to EPG simulation, a small decrease in signal intensity occurs from the center toward the edges of the profile for the later echoes (Figure 6A). The phase images showed a consistent quadratic phase in all echoes either with or without B_1_^+^‐dependent phase adjustment, but the quadratic phase acquired without B_1_^+^‐dependent phase adjustment had a slightly deeper quadratic phase at echo 1 as compared to the later echoes (Figure 6C) as is seen in EPG simulation (Figure 4A). Because the quadratic phase is consistent in all echoes, echo data can be combined without any special processing (e.g., removing the quadratic phase) in 3D FM‐FSE image reconstruction; thereby, the reconstructed image still retains the quadratic phase (Figure 6D).

**FIGURE 7:**
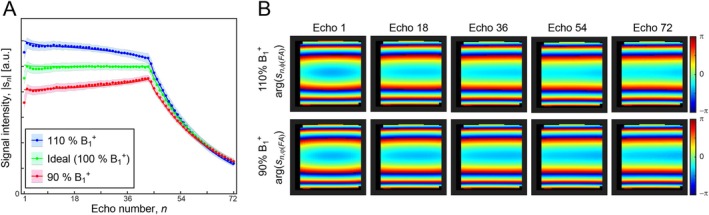
Experimental validation of the B_1_^+^ mismatch between the phase adjustment optimization and the actual B_1_^+^. Multi‐echo spin‐echo FM‐FSE acquisition was performed using ±10% of the ideal B_1_^+^ magnitude (90% or 110% B_1_^+^) by scaling the reference voltage. (A) The experimental data (mean ± standard deviation shown as points and shaded area) match well with the EPG simulation results (lines; Figure 4C,D). The echo signals are lower or higher than the ideal/100% B_1_^+^ setting with the 90% or 110% B_1_^+^ setting. While the ideal setting exhibits a flat echo signal until the flip angle reached the maximum, the 90% or 110% B_1_^+^ setting shows gradually increasing or decreasing echo signals. (B) The quadratic phase is consistently seen either with the 90% or 110% B_1_^+^ setting, which is highly comparable to the ideal case (Figure 6C).

To test the sensitivity to B_1_^+^ inhomogeneity, MESE measurements were performed by scaling the reference voltage by ±10% (90% and 110% B_1_^+^; Figure 7). The echo signal intensities in the MESE measurements matched well with the EPG simulation results (Figure 4C,D); the echo signal from the 90% B_1_^+^ setting was lower at echo 1 compared to the ideal setting (100% B_1_^+^) and then it gradually increased until the flip angle reached the maximum (Figure 7A). Conversely, the echo signal acquired with 110% B_1_^+^ was higher than the ideal setting at echo 1 and then it gradually decreased as the echo number increased. However, the quadratic phase is highly consistent for all three settings (Figures 6C and 7B).

### 4.4 In Vivo Human Brain Imaging

**FIGURE 8:**
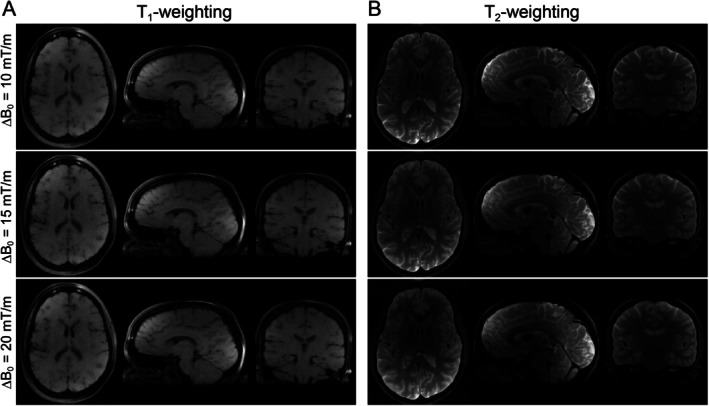
In vivo human brain images of 3D FM‐FSE with *T*_1_‐(A) and *T*_2_‐weighting (B). A linear gradient of 10/15/20 mT/m along the z axis was applied as an inhomogeneous field during the entire 3D FM‐FSE scan. Image contrasts are comparable regardless of the linear inhomogeneous fields for either *T*_1_‐ or *T*_2_‐weighted images.

In vivo human brain imaging was performed with a linear *Δ*B_0_ field inhomogeneity of 10, 15, and 20 mT/m along the z axis (Figure 8). Image contrasts were similar regardless of the *Δ*B_0_ field inhomogeneity in either *T*_1_‐ or *T*_2_‐weighted images. The developed 3D FM‐FSE worked robustly for all subjects (Figure S2).

**FIGURE 9:**
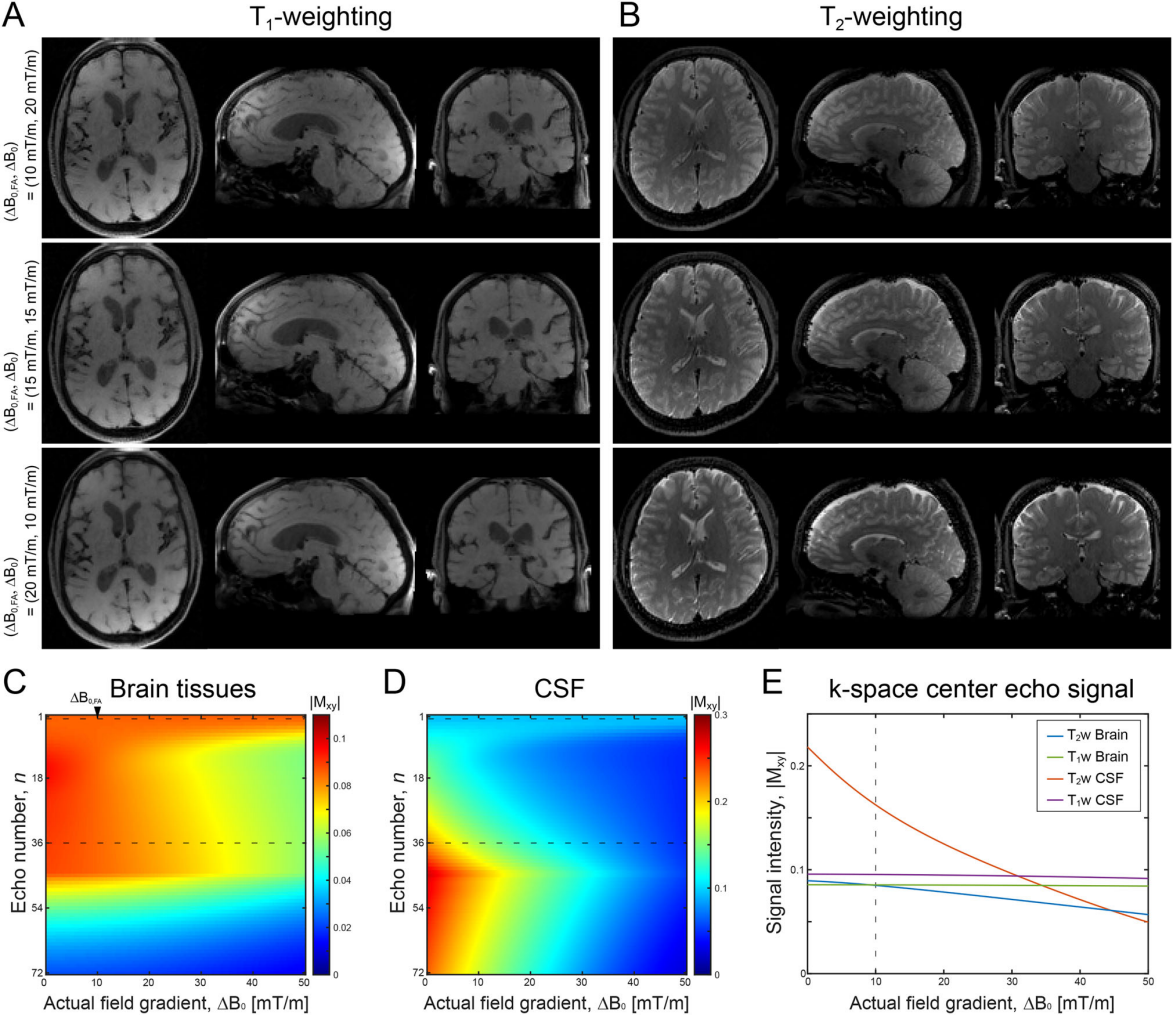
Impacts of the mismatch between the field gradients assumed in the flip angle calculation (*Δ*B_0,FA_) and in the actual inhomogeneous field (*Δ*B_0_ = 10/15/20 mT/m) in *T*_1_‐ (A) and (B) *T*_2_‐weighted brain images. Two largest mismatch cases, (*Δ*B_0,FA_, *Δ*B_0_) = (10 mT/m, 20 mT/m) and (20 mT/m, 10 mT/m) (top and bottom row), are shown along with one matched case, (15 mT/m, 15 mT/m) (center row). In *T*_1_‐weighted images, image contrasts were similar regardless of the gradient field mismatch (A). *T*_2_‐weighted images show comparable *T*_2_‐weighting in brain tissues (gray and white matters), but cerebrospinal fluid (CSF) shows visually recognizable intensity changes (B); when the assumed field gradient in the flip angle calculation is lower than the actual local field gradient (10 mT/m, 20 mT/m), there is stronger signal attenuation (top row) observed in CSF as compared to the matched case (center row), and vice versa (bottom row). EPG simulations of brain tissues (C) and CSF (D) with *Δ*B_0,FA_ = 10 mT/m and various field gradient *Δ*B_0_ (0–50 mT/m) supported the findings. The *k*‐space center echo in *T*_1_‐weighted imaging (*T*_1_w, echo 2) shows nearly constant signal intensities regardless of the actual field gradient, *Δ*B_0_, for either brain tissues or CSF (E). The *k*‐space center echo in *T*_2_‐weighted imaging (*T*_2_w, echo 36) shows signal intensity attenuation along with an increase of *Δ*B_0_ for either brain tissues or CSF. However, the CSF signal attenuation is much more sensitive to *Δ*B_0_ than brain tissues; when *Δ*B_0_ increased from 10 to 20 mT/m (i.e., top row in B), the *k*‐space center echo signal for brain tissues showed a 7.6% signal intensity attenuation, whereas the signal intensity attenuation was 23.1% for CSF because of its higher ADC value.

Because MRI systems with inhomogeneous magnetic fields typically exhibit nonlinear field distributions in space, the impact of discrepancies between the *Δ*B_0_ assumed in the flip angle calculation (*Δ*B_0,FA_) and the actual local field gradient (*Δ*B_0_) was tested under conditions where the two were not aligned (Figure 9A,B). In *T*_1_‐weighted images, there was no clear impact from the mismatch of *Δ*B_0,FA_ and *Δ*B_0_. In *T*_2_‐weighted images, while the *T*_2_ contrasts between gray and white matter were similar either with or without the *Δ*B_0_ mismatch, CSF showed visually clear intensity changes due to its higher ADC value. When *Δ*B_0,FA_ was higher than *Δ*B_0_, CSF showed high signal intensities; conversely, when *Δ*B_0,FA_ was lower than *Δ*B_0_, CSF was depicted with low signal intensities. The findings were validated by EPG simulations for brain tissues and CSF (Figure 9C,D). Signal intensities of the *k*‐space center echo in *T*_1_‐weighted imaging (echo 2) were nearly constant regardless of the actual field gradient, *Δ*B_0_, either for brain tissues or CSF. As such, image contrasts in the *T*_1_‐weighted images were highly consistent either with or without the *Δ*B_0_ mismatch. The *k*‐space center echo in *T*_2_‐weighted imaging (echo 36) shows signal intensity attenuation along with an increase of *Δ*B_0_ for brain tissues and CSF, but the CSF signal attenuation is much more sensitive to *Δ*B_0_ than brain tissues due to its higher ADC value. Consequently, CSF shows more conspicuous signal intensity changes with the *Δ*B_0_ mismatch as compared to brain tissues in the *T*_2_‐weighted images. The *k*‐space center echo signal attenuated by 7.6% for brain tissues when increasing *Δ*B_0_ from 10 to 20 mT/m, whereas CSF showed a 23% signal attenuation. Based on the EPG simulation, *Δ*B_0_ = 38 mT/m is required for brain tissues to generate the diffusion signal attenuation comparable to CSF. The impacts of the *Δ*B_0_ mismatch on diffusion signal attenuation were comparable in all three flip angle settings (*Δ*B_0,FA_ = 10/15/20 mT/m, Figure S3).

### 4.5 3D FM‐FSE in a Nonlinear Inhomogeneous Field

**FIGURE 10:**
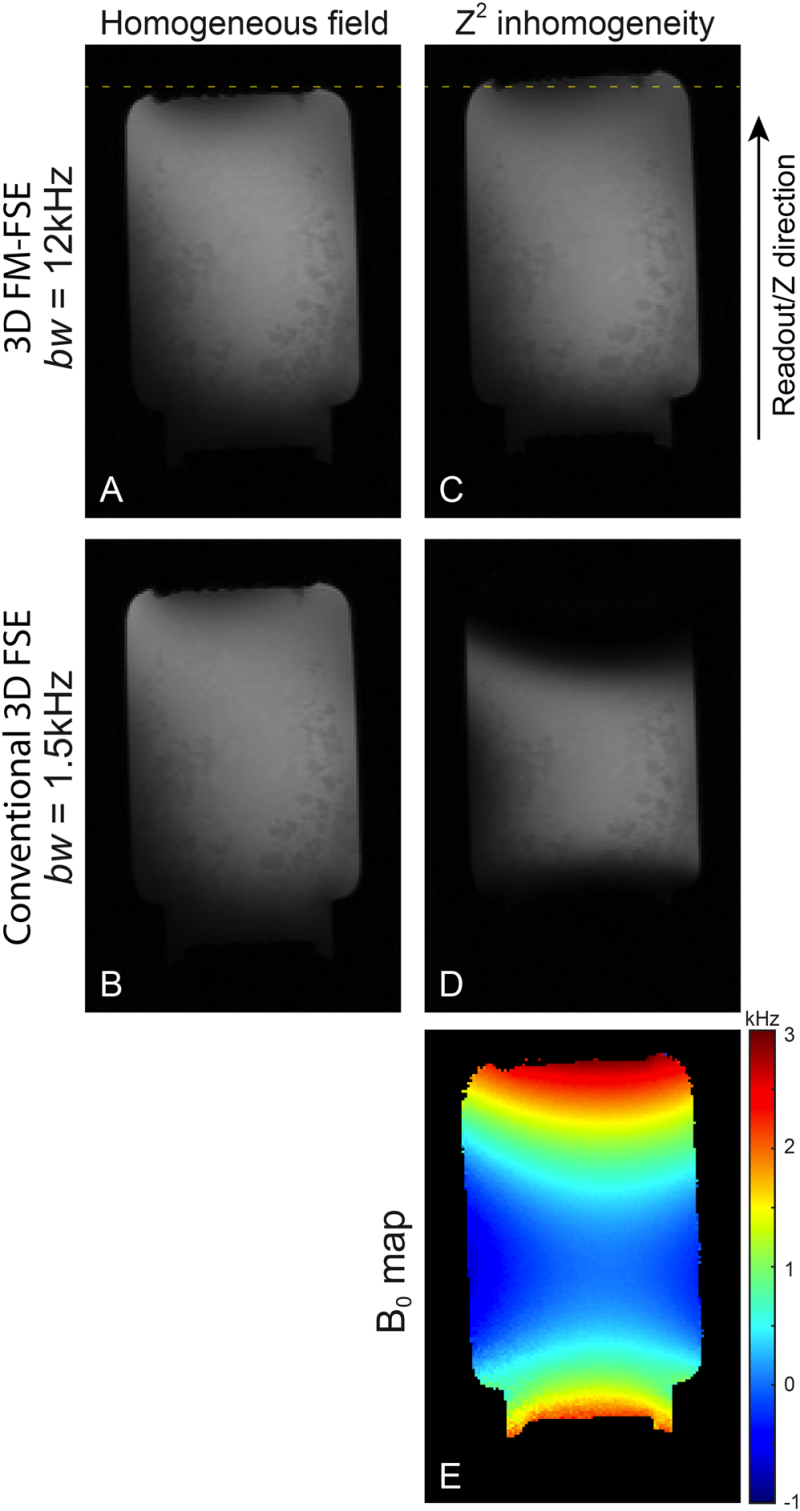
Comparison of 3D FM‐FSE and conventional 3D FSE in a nonlinear inhomogeneous field for an agarose gel phantom. Without field inhomogeneity, the 3D FM‐FSE image is nearly identical to the conventional 3D FSE image (A,B, left column). A nonlinear inhomogeneous field was introduced by adjusting the Z^2^ shim value to its maximum (C‐E, right column). Although the 3D FM‐FSE image shows image distortion along the readout/Z direction due to disturbance of the frequency encoding field (dashed yellow line in A and C), the image is comparable to that acquired without the nonlinear field inhomogeneity. With conventional 3D FSE with hard pulse excitation and refocusing, only spins within the hard pulse bandwidth (1.5 kHz) were imaged, and therefore the regions outside of the ±750 Hz frequency range were not visualized (D,E).

A nonlinear inhomogeneous magnetic field was introduced by adjusting the Z^2^ shim value to its maximum. Without the Z^2^ field inhomogeneity (i.e., in a homogeneous field), 3D FM‐FSE and conventional FSE provided nearly identical images (Figure 10A,B). With the Z^2^ field inhomogeneity, although the 3D FM‐FSE image shows image distortion along the readout/frequency encoding dimension because of interference of the readout gradient field and the Z^2^ inhomogeneous field, the entire area of the agarose gel phantom was imaged (Figure 10C). However, only a portion of the phantom was visualized with the conventional 3D FSE sequence, because the hard pulse used for non‐selective excitation and refocusing had only 1.5‐kHz bandwidth and as such spins outside of the bandwidth were not excited or refocused (Figure 10D,E).

## 5 Discussion

The condition to generate a consistent quadratic phase profile in even and odd refocused echoes in 3D FM‐FSE has been formulated using Cayley‐Klein parameters. The FM pulses used for refocusing were applied in the non‐adiabatic regime; thereby, variable flip angles were implemented to achieve a long echo train length, as is done in clinical 3D FSE sequences [25, 34, 37]. The B_1_‐dependent phase in FM pulse excitation and refocusing was compensated by introducing a B_1_^+^‐dependent adjustment of the initial phase of the FM pulses in the 3D FM‐FSE sequence. The expected robust performance of the proposed 3D FM‐FSE sequence was demonstrated with a clinical 3 T MRI system with a linear z inhomogeneous field gradient and a nonlinear inhomogeneous field introduced by intentionally adjusting the Z^2^ shim value in the B_0_‐shim coil set.

The consistent quadratic phase condition demonstrated herein does not remove the quadratic phase from FM pulses, but instead makes it consistent in even and odd refocused echo components. As such, the quadratic phase in the readout direction is retained in the reconstructed 3D images. While the quadratic phase profile results in signal cancelation along the slice‐selective dimension in 2D imaging, it does not cause issues in 3D imaging as long as the spin magnetizations within the FM pulse bandwidth are refocused during readout. This requires the matrix size in the readout dimension to be larger than r·TBP ($=T_{l,}\cdot \mathrm{bw}$; Equations (3) and (4)); that is, 18.8 points in readout with the HS2 pulse used herein. This requirement is readily satisfied in most cases.

The B_1_^+^‐dependent phase was determined using only the *β* parameter, because the phase from the *α* parameter is minor and thus can be ignored. However, the *α* parameter exhibits some B_1_^+^‐dependent phase near the profile edges as B_1_^+^/FA increases (Figure S1B,C). Because the *α* phase is an odd function with respect to resonance offset and increases toward the profile edges, even without the B_1_^+^‐dependent phase adjustment, the B_1_^+^‐dependence of the *α* parameter compensated the B_1_^+^‐dependent phase toward one edge of the profile, but increased phase error occurs toward the other edge, resulting in the observed slanted magnitude profile at later echoes (Figures 4A and 6A,B). The B_1_^+^‐dependent phase adjustment perfectly compensated the phase errors at the center isochromat, but the B_1_^+^‐dependent phase in the *α* parameter introduced minor phase errors around the profile edges, which resulted in slight intensity drops equally in both edges of the profile (Figures 4B and 6A).

In this study, we focused on the CPMG phase condition in the FSE echo train because it is the most efficient condition to preserve the refocused echo signals in FSE. However, when the CPMG condition is disrupted, for example, by subtle motion in inhomogeneous magnetic fields, it is well known that the non‐CPMG component rapidly decays away in the FSE echo train [38]. To avoid the discrepancy in the echo train signal decay between the CPMG and non‐CPMG components, 0–π/2 phase cycling was introduced in the refocusing RF pulses in FSE for diffusion MRI [39]. Such modification in phase cycling of the refocusing RF pulses may make 3D FM‐FSE more robust to motion. In this case, even and odd echoes represent different phase profiles depending on the initial phase of the transverse magnetization. As such, extra modifications in the pulse sequence and image reconstruction are required to reconstruct a single image from even and odd echoes in 3D FM‐FSE. This will be explored in future studies.

In this study, we tested the linear inhomogeneous field and the Z^2^‐shape nonlinear inhomogeneous field. Although the Z^2^ inhomogeneous field tested herein is relatively minor as compared to the readout gradient field, nonlinear inhomogeneous fields typically lead to geometric and intensity distortions in reconstructed images (i.e., spatially varying resolution and SNR). However, when the inhomogeneous field distribution is known, image distortion can be easily corrected in a similar way to the method used to correct image distortion associated with gradient nonlinearity [3, 40, 41]. Another adverse effect of the nonlinear field inhomogeneity is spatially varying diffusion weighting. While there was no conspicuous impact observed in brain tissues with inhomogeneous field gradients in a range of 10–20 mT/m, the CSF signal intensities in *T*_2_‐weighted images decreased as the field gradient increased due to the high ADC value. The limitation from the nonlinear field inhomogeneity is practically determined by a combination of these two factors; a steep local field gradient results in lower SNR both in image distortion and diffusion weighting.

In this study, we demonstrated the capability of 3D FM‐FSE for MRI in inhomogeneous magnetic fields, where FM pulses were used for broadband excitation and refocusing to cover the broadly distributed frequency distribution in inhomogeneous magnetic fields. However, the field inhomogeneities intentionally introduced in this study are far more than those in current clinical settings. Significant magnetic field inhomogeneity is one of the major difficulties in compact and portable MRI systems which have recently been developed actively to broaden the accessibility and clinical utility of MRI across diverse healthcare settings [42, 43]. The proposed 3D FM‐FSE sequence can be a potential imaging method in such compact MRI systems.

## 6 Conclusion

A 3D FM‐FSE sequence has been introduced to enable MRI scanning in a highly inhomogeneous magnetic field. In 3D FM‐FSE, a consistent quadratic phase is generated in both even and odd refocused echo components, and as such all refocused echoes in FSE can be used for 3D imaging without any special processing on the quadratic phase. The proposed FM‐FSE sequence can be a promising 3D imaging pulse sequence in inhomogeneous static magnetic fields.

## Funding

This work was supported by the National Institutes of Health (grant nos. P41EB027061, S10OD017974, U01EB025153).

## Supporting information


**Figure S1.** B_1_^+^‐dependent phase in the Cayley‐Klein parameters (*α, β*)^T^ (left and center columns) and excitation profile (*M*_xy_ = 2α*β) (right column) for a flattened hyperbolic secant (HS2) pulse. Magnitude (A) and phase (B) are plotted with respect to flip angle in a range of [0°, 120°]. While the phase of *α* is mostly flat especially with low flip angles, the quadratic phase in the excitation profile is predominantly attributed to the phase of *β*. The phase of *α* at the center isochromat is zero regardless of the flip angle (C). The B_1_^+^/flip‐angle‐dependent phase offset in the excitation profile is predominantly contributed by the *β* parameter. Therefore, the B_1_^+^ dependent phase offset, ϕ(FA), is given by $\arg\left(M_{\mathrm{xy}}\right)=\arg\left(\beta \right)$ for the center isochromat. The *α* phase also shows B_1_^+^‐dependence as it gets close to the profile edges.
**Figure S2.** 3D FM‐FSE brain images for eight of ten volunteer subjects. The images were acquired with *T*_2_‐weighted contrasts and a linear inhomogeneous gradient field of *Δ*B_0_ = 15 mT/m. The images from the remaining two subjects are shown in Figures 8B and 9B.
**Figure S3.** EPG simulation of the *Δ*B_0_ mismatch between flip angle optimization, *Δ*B_0,FA_, and actual field gradient, *Δ*B_0_, for the flip angle array optimized with *Δ*B_0,FA_ = 15 mT/m (A–C) and *Δ*B_0,FA_ = 20 mT/m (D–F). The impacts of the *Δ*B_0_ mismatch are similar to the case with the flip angle array optimized with *Δ*B_0,FA_ = 10 mT/m (Figure 9C–E) for both *Δ*B_0,FA_ = 15 mT/m and *Δ*B_0,FA_ = 20 mT/m. The *k*‐space center echo signals in *T*_1_‐weighted imaging (echo 2, *T*_1_w) are nearly constant regardless of the actual field gradient *Δ*B_0_ for either brain tissues or CSF. The *k*‐space center echo signals in *T*_2_‐weighted imaging (echo 36, *T*_2_w) attenuated along with an increase of *Δ*B_0_ for brain tissues and CSF. When *Δ*B_0_ was increased from 10 to 20 mT/m, attenuation of the *k*‐space center echo signal in *T*_2_w was 7.1% and 22.7% for brain tissues and CSF with *Δ*B_0,FA_ = 15 mT/m, and 6.6% and 22.2% for brain tissues and CSF with *Δ*B_0,FA_ = 20 mT/m, respectively. These signal attenuations are comparable to those with *Δ*B_0,FA_ = 10 mT/m (7.6% and 23.1%, Figure 9E).

## Data Availability

The data that support the findings of this study are available from the corresponding author upon reasonable request.
