# Supplementary material for: Consistent Quadratic Phase Formation in 3D Fast Spin Echo Using Frequency‐Modulated RF Pulses
Source: Magn Reson Med. 2026 Apr 2;96(2):666–81. doi: 10.1002/mrm.70368 (PMC13269193; doi:10.1002/mrm.70368)
Supplement: Supplementary file 1 — Figure S1. B1 +‐dependent phase in the Cayley‐Klein parameters (α, β)T (left and center columns) and excitation profile (M xy = 2α*β) (right column) for a flattened hyperbolic secant (HS2) pulse. Magnitude (A) and phase (B) are plotted with respect to flip angle in a range of [0°, 120°]. While the phase of α is mostly flat especially with low flip angles, the quadratic phase in the excitation profile is predominantly attributed to the phase of β. The phase of α at the center isochromat is zero regardless of the flip angle (C). The B1 +/flip‐angle‐dependent phase offset in the excitation profile is predominantly contributed by the β parameter. Therefore, the B1 + dependent phase offset, ϕ(FA), is given by argMxy=arg(β) for the center isochromat. The α phase also shows B1 +‐dependence as it gets close to the profile edges. Figure S2. 3D FM‐FSE brain images for eight of ten volunteer subjects. The images were acquired with T 2‐weighted contrasts and a linear inhomogeneous gradient field of ΔB0 = 15 mT/m. The images from the remaining two subjects are shown in Figures 8B and 9B. Figure S3. EPG simulation of the ΔB0 mismatch between flip angle optimization, ΔB0,FA, and actual field gradient, ΔB0, for the flip angle array optimized with ΔB0,FA = 15 mT/m (A–C) and ΔB0,FA = 20 mT/m (D–F). The impacts of the ΔB0 mismatch are similar to the case with the flip angle array optimized with ΔB0,FA = 10 mT/m (Figure 9C–E) for both ΔB0,FA = 15 mT/m and ΔB0,FA = 20 mT/m. The k‐space center echo signals in T 1‐weighted imaging (echo 2, T 1w) are nearly constant regardless of the actual field gradient ΔB0 for either brain tissues or CSF. The k‐space center echo signals in T 2‐weighted imaging (echo 36, T 2w) attenuated along with an increase of ΔB0 for brain tissues and CSF. When ΔB0 was increased from 10 to 20 mT/m, attenuation of the k‐space center echo signal in T 2w was 7.1% and 22.7% for brain tissues and CSF with ΔB0,FA = 15 mT/m, and 6.6% and 22.2% for brain tissues and CSF with [file MRM-96-666-s001.docx]

**Supplemental material**

**
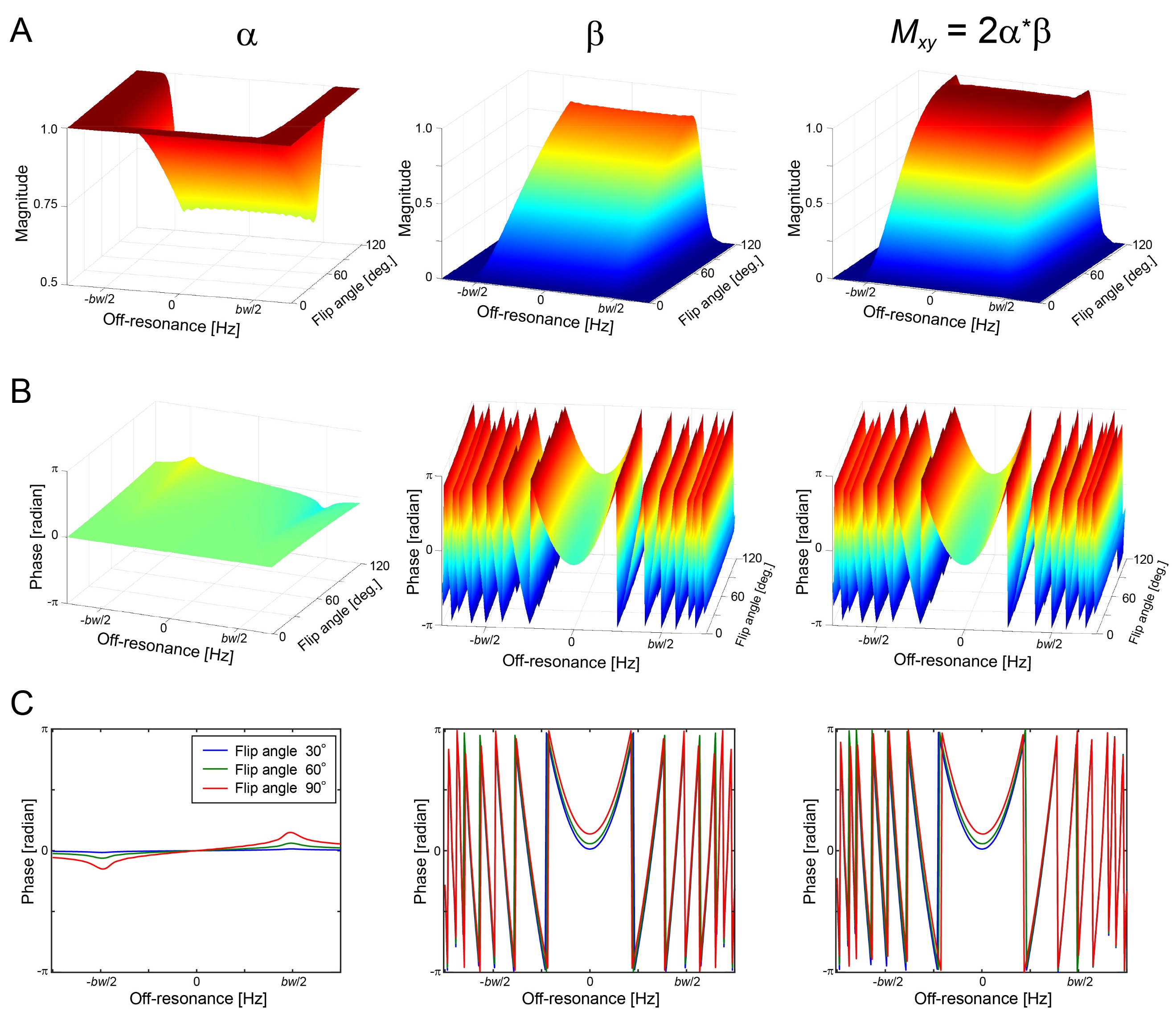
**

**Figure S1**. B_1_^+^-dependent phase in the Cayley-Klein parameters (*α, β*)^T^ (left and center columns) and excitation profile (M_xy_ = 2*α*^*^β) (right column) for a flattened hyperbolic secant (HS2) pulse. Magnitude (A) and phase (B) are plotted with respect to flip angle in a range of [0°, 120°]. While the phase of *α* is mostly flat especially with low flip angles, the quadratic phase in the excitation profile is predominantly attributed to the phase of *β*. The phase of *α* at the center isochromat is zero regardless of the flip angle (C). The B_1_^+^/flip-angle-dependent phase offset in the excitation profile is predominantly contributed by the *β* parameter. Therefore, the B_1_^+^ dependent phase offset, $\phi\left( FA \right)$, is given by $\arg(M_{xy})=arg(\beta)$ for the center isochromat. The *α* phase also shows B_1_^+^-dependence as it gets close to the profile edges.


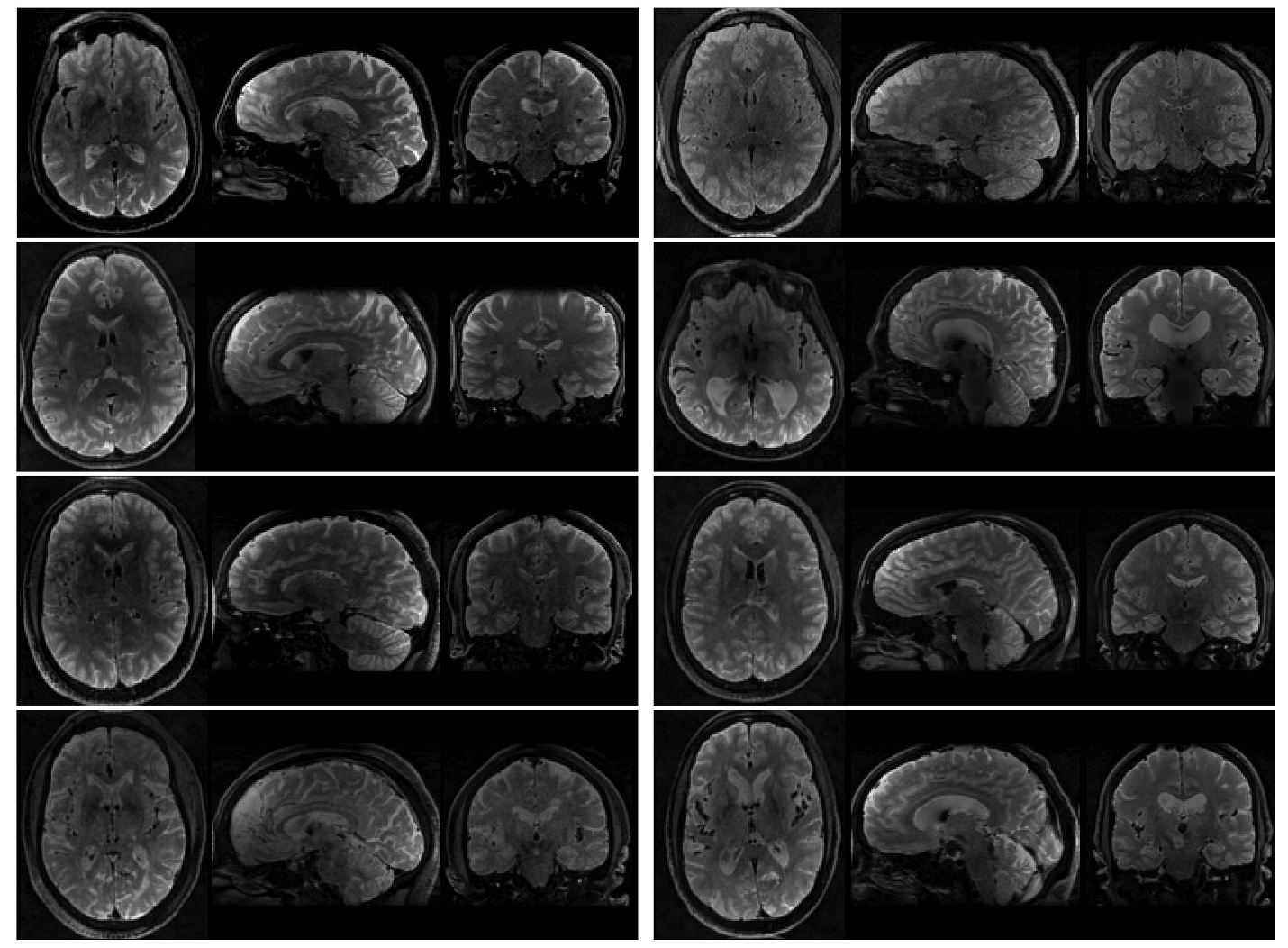


**Figure S2**. 3D FM-FSE brain images for eight of ten volunteer subjects. The images were acquired with T_2_-weighted contrasts and a linear inhomogeneous gradient field of *Δ*B_0_ = 15 mT/m. The images from the remaining two subjects are shown in Figures 8B and 9B.


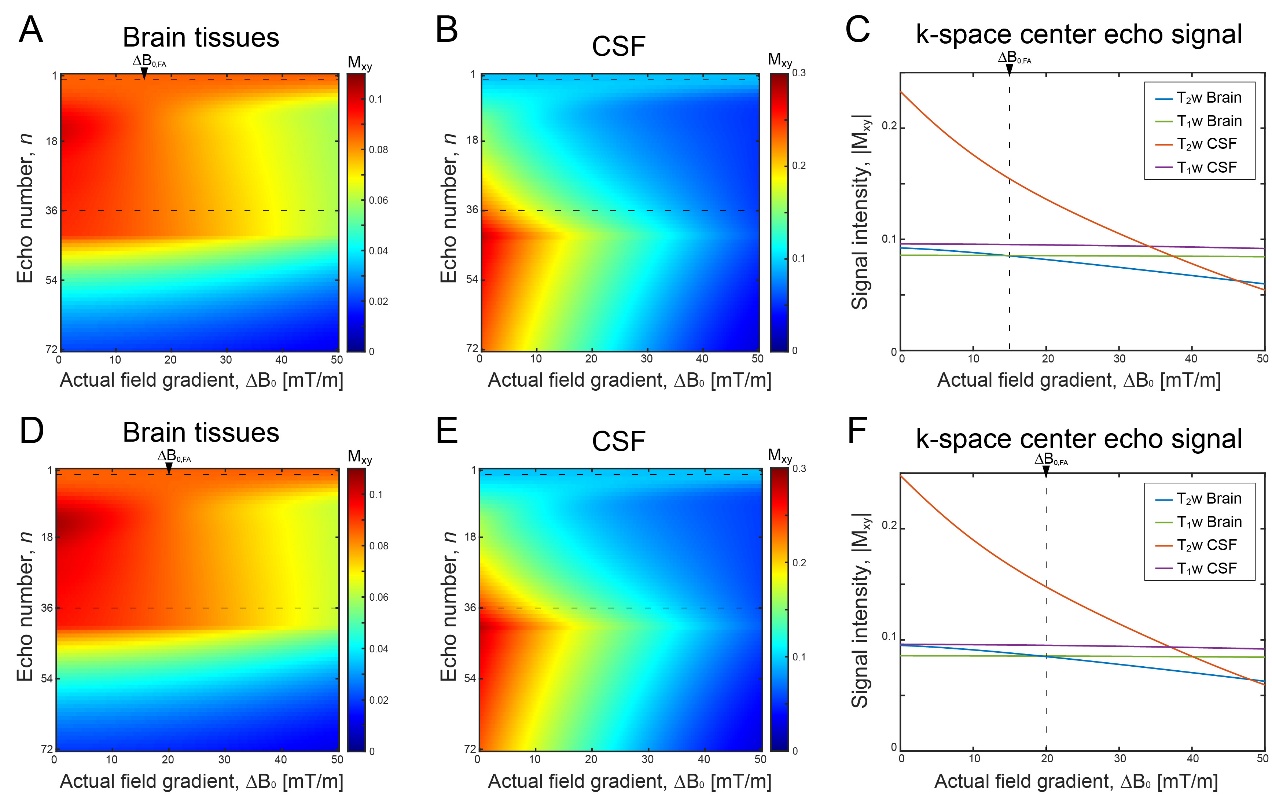


**Figure S3**. EPG simulation of the *Δ*B_0_ mismatch between flip angle optimization, *Δ*B_0,FA_, and actual field gradient, *Δ*B_0_, for the flip angle array optimized with *Δ*B_0,FA_ = 15 mT/m (A-C) and *Δ*B_0,FA_ = 20 mT/m (D-F). The impacts of the *Δ*B_0_ mismatch are similar to the case with the flip angle array optimized with *Δ*B_0,FA_ = 10 mT/m (Figure 9C-E) for both *Δ*B_0,FA_ = 15 mT/m and *Δ*B_0,FA_ = 20 mT/m. The k-space center echo signals in T_1_-weighted imaging (echo 2, T_1_w) are nearly constant regardless of the actual field gradient *Δ*B_0_ for either brain tissues or CSF. The k-space center echo signals in T_2_-weighted imaging (echo 36, T_2_w) attenuated along with an increase of *Δ*B_0_ for brain tissues and CSF. When *Δ*B_0_ was increased from 10 mT/m to 20 mT/m, attenuation of the k-space center echo signal in T_2_w was 7.1% and 22.7% for brain tissues and CSF with *Δ*B_0,FA_ = 15 mT/m, and 6.6% and 22.2% for brain tissues and CSF with *Δ*B_0,FA_ = 20 mT/m, respectively. These signal attenuations are comparable to those with *Δ*B_0,FA_ = 10 mT/m (7.6% and 23.1%, Figure 9E).
